# *Fasciola hepatica* reinfection potentiates a mixed Th1/Th2/Th17/Treg response and correlates with the clinical phenotypes of anemia

**DOI:** 10.1371/journal.pone.0173456

**Published:** 2017-03-31

**Authors:** M. Adela Valero, Ignacio Perez-Crespo, Carlos Chillón-Marinas, Messaoud Khoubbane, Carla Quesada, Marta Reguera-Gomez, Santiago Mas-Coma, Manuel Fresno, Núria Gironès

**Affiliations:** 1 Departamento de Parasitología, Facultad de Farmacia, Universidad de Valencia, Av. Vicent Andrés Estellés s/n, Burjassot, Valencia, Spain; 2 Centro de Biología Molecular “Severo Ochoa”, Consejo Superior de Investigaciones Científicas (CSIC)-Universidad Autónoma de Madrid (UAM), C/ Nicolás Cabrera 1, Cantoblanco, Madrid, Spain; Centro de Investigacion y de Estudios Avanzados del Instituto Politecnico Nacional, MEXICO

## Abstract

**Background:**

Fascioliasis is a severe zoonotic disease of worldwide extension caused by liver flukes. In human fascioliasis hyperendemic areas, reinfection and chronicity are the norm and anemia is the main sign. Herein, the profile of the Th1/Th2/Th17/Treg expression levels is analyzed after reinfection, correlating them with their corresponding hematological biomarkers of morbidity.

**Methodology/Principal findings:**

The experimental design reproduces the usual reinfection/chronicity conditions in human fascioliasis endemic areas and included *Fasciola hepatica* primo-infected Wistar rats (PI) and rats reinfected at 8 weeks (R8), and at 12 weeks (R12), and negative control rats. In a cross-sectional study, the expression of the genes associated with Th1 (*Ifng*, *Il12a*, *Il12b*, *Nos2)*, Th2 *(Il4*, *Arg1)*, Treg (*Foxp3*, *Il10*, *Tgfb*, *Ebi3)*, and Th17 (*Il17)* in the spleen and thymus was analyzed. After 20 weeks of primary infection, PI did not present significant changes in the expression of those genes when compared to non-infected rats (NI), but an increase of *Il4*, *Arg1* and *Ifng* mRNA in the spleen was observed in R12, suggesting the existence of an active mixed Th1/Th2 systemic immune response in reinfection. *Foxp3*, *Il10*, *Tgfb* and *Ebi3* levels increased in the spleen in R12 when compared to NI and PI, indicating that the Treg gene expression levels are potentiated in chronic phase reinfection. Il17 gene expression levels in R12 in the spleen increased when compared to NI, PI and R8. Gene expression levels of *Il10* in the thymus increased when compared to NI and PI in R12. *Ifng* expression levels in the thymus increased in all reinfected rats, but not in PI. The clinical phenotype was determined by the fluke burden, the rat body weight and the hemogram. Multivariate mathematical models were built to describe the Th1/Th2/Th17/Treg expression levels and the clinical phenotype. In reinfection, two phenotypic patterns were detected: i) one which includes only increased splenic *Ifng* expression levels but no Treg expression, correlating with severe anemia; ii) another which includes increased splenic *Ifng* and Treg expression levels, correlating with a less severe anemia.

**Conclusions/Significance:**

In animals with established *F*. *hepatica* infection a huge increase in the immune response occurs, being a mixed Th2/Treg associated gene expression together with an expression of *Ifng*. Interestingly, a Th17 associated gene expression is also observed. Reinfection in the chronic phase is able to activate a mixed immune response (Th1/Th2/Th17/Treg) against *F*. *hepatica* but T and B proliferation to mitogens is strongly suppressed in all infected rats vs control in the advanced chronic phase independently of reinfection The systemic immune response is different in each group, suggesting that suppression is mediated by different mechanisms in each case. Immune suppression could be due to the parasite in PI and R8 rats and the induction of suppressive cells such as Treg in R12. This is the first study to provide fundamental insight into the immune profile in fascioliasis reinfection and its relation with the clinical phenotypes of anemia.

## Introduction

As a consequence of the effects of climate as well as global changes, in general, prevalences, intensities and the geographical distribution of trematodiases have become a priority in public health [1]. Fascioliasis is a severe zoonotic disease caused by two trematode species, *Fasciola hepatica* and *F*. *gigantica*, infecting the liver of a wide range of mammals, mainly livestock worldwide [2]. The human fascioliasis scenario experienced a profound change in the 1990’s, from isolated cases from developed countries to the progressive description of heterogeneous human endemic areas and human infection reports in developing countries, presenting different transmission patterns and epidemiological situations [2]. The last stage of the disease in humans encompasses an obstructive or chronic phase which may develop after months to years of infection, including mild to moderate anemia, especially in cases of heavy parasitic burdens [3]. Therefore, human fascioliasis is included in the WHO list of priorities among foodborne trematodiases.

The risk of reinfection in infected humans in hyperendemic areas is very high. The frequent chronicity in infected subjects—the majority of whom are in the chronic phase of the disease, lasting for many years—is due to the fluke’s enormous longevity in humans (of up to 13.5 years) and due to the lack of diagnosis and treatment of patients, mainly children [2,4]. Indeed, chronicity is evidenced by recent multitest diagnostic studies, which have demonstrated that most of the children infected (from the age of five) in these endemic areas are already in the biliary or chronic phase of the disease [5], i.e., reinfection and chronicity are the norm in these areas.

Although little is known on fascioliasis reinfection in humans, it has been suggested to be linked to high burdens [2] and their impact during the chronic phase [3], and to coinfections with other diseases, whether parasitic [2] or bacterial [6], giving rise to a combined morbidity potential of concern in human fascioliasis high endemic areas. Additionally, reinfection has also been related to high pathogenicity and impressive manifestations in patients whether directly by immunologically stimulating the ectopic migration of invasive juvenile flukes or indirectly by increasing complex mechanisms involved in immuno-allergic and toxic processes caused by flukes in the liver but acting systemically at distance [4]. Clinical studies have shown this disease to be pronouncedly complex, giving rise to progressive general deterioration of the patients, with sequelae sometimes leaving subjects handicapped and frail, even leading to fatal cases [2,4,7].

Host immune responses are characterized by T-cell activation in response to antigen stimulation leading to the differentiation of effector T-cell subtypes, which, in turn, are characterized by distinct cytokine secretion and enzymatic profiles leading to specific effector functions. Helminths generally induce Th2 responses, which are characterized by IL-4 and IL-13 production [8], with alternative activation of macrophages expressing Arginase I (Arg I) [9]. Arg I may downregulate inflammatory responses and facilitate tissue remodeling, as it initiates the pathways that lead to the synthesis of proline and polyamines while competing with inducible nitric oxide synthase-2 (iNOS or NOS-2), an enzyme that converts L-arginine into nitric oxide (NO) [9]. Conversely, intracellular pathogens generate Th1 responses, induced by IL-12, composed of IL-12α and IL-12β, triggering IFN-γ production [10], which in turn induces classically activated macrophages, triggering iNOS expression. In addition, Th17 cells, characterized by IL-17 production, cause recrudescence of autoimmune disease [11]. Uncontrolled Th1 responses can lead to necrosis and tissue damage, whereas exaggerated responses by Th2 cells can induce asthma, as well as allergy and can also lead to tissue inflammation and fibrosis [12].

The immune balance and immunopathology control are mediated by regulatory CD4+CD25+ T cells (Tregs) [12] in the thymus, named natural Tregs (nTregs), which express the transcription factor forkhead box P3 (Foxp3). nTregs can reach the periphery as functional suppressor cells [13]. Tregs can also be induced in the periphery when the infection occurs (iTregs or Th3 cells) [14,15]. Tregs exert their suppressive function by producing IL-10 and TGF-β, or indirectly via dendritic cells [16,17]. IL-35 is an inhibitory cytokine that may be specifically produced by Foxp3+ Treg cells and is required for maximal suppressive activity, composed by IL-12α (Il12a) and IL-27β (Ebi3). Tr1 cells are iTregs that produce IL-10 but do not express Foxp3 [16]. In some settings a complex network of both Foxp3+ Tregs and combinations of IL-10 and TGF-β has been found to play an important role in controlling host immune responses, thereby preventing parasite death and resolution of infection. However, reversal of these regulatory settings has been shown to result in parasite clearance [18].

A strong Th2 immune response is induced in most helminth infections with early production of IL-4 over IFN-γ, playing a dominant role in host protective immunity and being related to the decrease in worm burdens as well as disease severity [19]. Most nematode infections follow this rule, while there are exceptions in the case of trematode infections such as *Schistosoma* species, in which both Th1 and Th2 responses have been associated with protection [20,21]. In fascioliasis, it has been reported that in the early process of infection (tissue or biliary canal habitat), *F*. *hepatica* induces potent polarized Th2/Treg immune responses coincident with a suppression of Th1/Th17 cytokines [22–27]. Previous studies showed that fascioliasis stimulates a switch to the Th2 immune response in the acute and chronic phases of primoinfection in several animal models [25,28,29]. In murine and ruminant models, soon after infection with *F*. *hepatica*, the immune response is proinflammatory, lasting about 4–6 weeks. This immune response is switched off at around the time the adult flukes begin to enter the bile duct [30]. Furthermore, infection also results in the bystander suppression of Th1 responses to a concurrent bacterial infection or to immunization with a Th1-inducing bacterial vaccine [31]. Little light has hitherto been shed on the regulation of *F*. *hepatica* immune responses. However, the existence of parasite specific, IL-10 and TGF-β producing, Treg cells capable of suppressing parasite-specific Th1 and Th2 responses has been demonstrated in a mice model [32]. Nevertheless, the few immunological studies on fascioliasis reinfection previously performed in animal models are old and outdated as a Th1/Th2/Th17/Treg analysis was not carried out.

Fascioliasis shows a marked variability in its immune response [33]. For instance, *R*. *norvegicus* Wistar has been classified, like humans, as a resistant host for *F*. *hepatica* [3,6,34,35]. Therefore, the Wistar rat model is considered a useful approach for the immunopathological research of fascioliasis, as the rat’s resistance level, susceptibility and pathology closely mimic chronic disease in humans. In this *F*. *hepatica*/Wistar model, during early chronic primoinfection, there is a predominance of a Th2 response, which decreases in advanced chronic infection (20 weeks post-infection, w.p.i.) characterized by a persistent immune suppression [35].

The aim of this study was to ascertain the immune response induced by reinfection during the chronic phase of *F*. *hepatica* infection by analyzing the immune profile through the gene expression of *Ifng*, *Ebi3*, *Nos2*, *Il4*, *Arg1*, *Foxp3*, *Il10*, *Tgfb and Il17* in the spleen and the thymus. The experimental design used reproduces the usual reinfection/chronicity conditions in human endemic areas and included primoinfected (PI) and reinfected rats at 8 weeks post-infection (w.p.i., R8), approximately the time when adult flukes begin to enter the bile duct or the initial chronic phase, and at 12 w.p.i (R12), when the adult flukes are already established in the bile duct (established chronic phase). The immune response in advanced chronic fascioliasis was analyzed at 20 weeks after primoinfection. Hitherto, the correlation between fascioliasis reinfection, anemia and the immune response has never been studied in a rat model at such an advanced chronic phase and during such a long time-span of 20 weeks post-infection (p.i.). The results revealed that reinfection reactivated the Th2 responses when compared to PI rats. In addition, we found, for the first time, that reinfection also stimulated Th1, Treg and Th17 responses. Finally, the correlation of the presence/absence of anemia and the variation of the different hematological parameters are correlated with the Th1/Th2/Th17/Treg associated gene expression levels.

## Materials and methods

### Materials

An *F*. *hepatica* isolate and lymnaeid snail vectors from a human fascioliasis endemic area were used. A balanced commercial rodent diet (Panlab Chow A04) and water were provided *ad libitum*. Metacercariae were obtained from experimentally infected *Galba truncatula* snails at the Department of Parasitology at University of Valencia, stored in freshwater at 4°C until required and administered to the rats after checking for viability by use of the refractile appearance of the excretory granules as a criterion. *Galba truncatula* that shed the cercariae that gave rise to the metacercariae were from a laboratory-reared strain (in Heraeus-Vötsch HPS 1500 and HPS 500 climatic chambers; the experimental conditions were as follows: temperature, 20°C; photoperiod, 12 h of light and 12 h of darkness; and relative humidity, 90%). These snails were, in turn, monomiracidially infected [36]. Male Wistar rats (80–100 g) were infected with 20 metacercariae/rat, by use of an orogastric syringe. First, a pilot experiment was conducted. Data from this experiment were used to calculate the number of animals of the experiment. *Fasciola* infection is harmful to rats and a long-term experiment was planned, therefore the software Ene 3.0 (Servei d’Estadistica Aplicada, Universidad Autonoma de Barcelona, Cerdanyola del Valles, Spain, distributed by GlaxoSmithKline) was used to establish the minimum number of animals needed to obtain statistically significant results (P: 0.05, power: 80%), in accordance with the present ethical rules for experiments with mammal animal models. Animal care, animal health, body condition and well-being were assessed on a weekly basis by means of checking their body weight and the appearance of the fur. Primoinfected and reinfected animals presented a lower body weight than negative controls at the end of the experiment. No mortality occurred. At the end of experiment, animals were anesthetized with an anesthetic (IsoFlo; Dr Esteve SA, Barcelona, Spain), prior to cardiac puncture for blood sample collection. Finally, animals were humanely euthanized with an overdose of the anesthetic. The number of worms that successfully developed in each rat was established by necropsy. Then, both the thymus and spleen were removed under sterile conditions.

### Ethics statement

All animal research was performed with the approval of the Committee for the Evaluation of Projects concerning Animal Research at University of Valencia (“Organo Habilitado para la Evaluación de Proyectos de Experimentación Animal de la Universidad de Valencia”) (A1263915389140 and 2015/VSC/PEA/00001 tipo 2), strictly following the institution’s guidelines based on Directive 2010/63/EU.

### Experimental infection

**Fig 1:**
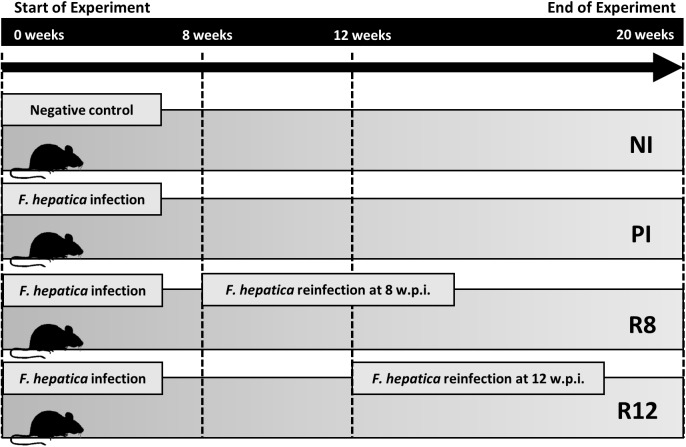
Experimental Design. Primary infected rats (PI): received a single infection dose; reinfected rats (R): received a primary infection and subsequently a secondary infection, at 8 weeks after primary infection (R8) and at 12 weeks after primary infection (R12); control rats (NI); all rats were sacrificed 20 weeks after initiation of the experiment.

The experimental design is depicted in Fig 1. Rats were divided into four groups: i) the non-infected group (NI) (16 rats); ii) a group that received a single infection dose (PI) (12 rats); iii) primary infected rats subsequently reinfected at 8 weeks (R8) group (14 rats); and iv) primary infected rats subsequently reinfected at 12 weeks p.i. (R12) group (16 rats). All rats were euthanized 20 weeks after initiation of the experiment.

### Quantitative RT-PCR gene expression

Total mRNA was extracted from the spleen and thymus. The isolated total mRNA was suspended in DEPC water for storage at −70°C and cDNA synthesis was performed with First Strand cDNA synthesis kit purchased from Pharmacia Biotech. The amplification of *Ifng*, *Nos2*, *Il4*, *Arg1*, *Foxp3*, *Il10*, *Tgfb*, *Ebi3*, *Il12a*, *Il12b*, *Il17* and *18S* cDNAs was performed in 384-well MicroAmp optical reaction plates using commercially available primer and probe sequences (TaqMan Gene Expression Assay reagents) and Universal PCR Master Mix (all from Applied Biosystems, Forter City, CA, USA). The total reaction volume in each well was 10 μl containing 5 ng of cDNA. The reactions were carried out in AB7900 HT Sequence Detection System (Applied Biosystems) using the standard three-step run protocol (Step 1: 2 min at 50°C, Step 2: 10 min at 95°C, Step 2: 40 cycles of 15 s at 95°C plus 1 min at 60°C). Quantification was calculated by the comparative threshold cycle (C_T_) method following the manufacturer’s instructions. All quantifications were normalized to the *18S* gene to account for the variability in the initial mRNA concentration the conversion efficiency of the reverse transcription reaction (ΔC_T_) as well as values from control samples from non-infected rats (ΔΔC_T_). The relative quantity (RQ) was calculated as: RQ = 2^-ΔΔCT^. SDS 2.2.2 software was used for analysis.

### Proliferation assays

In the advanced (20 w.p.i.) chronic phase, spleens from NI, PI, R8 and R12 rats were extracted, and cells were obtained using a 40-μm mesh cell strainer (Becton Dickinson Labware). After red blood cells were lysed with water for 5 s, the cells were washed twice in Dulbecco’s modified Eagle medium (DMEM) and resuspended in DMEM 10% fetal bovine serum (FBS). Cells were plated in 96-well plates (2–105 cells/200 μL) containing 5 μg/mL of concanavalin A (ConA) and 1 μg/ml of lipopolysaccharide (LPS), as indicated. Proliferation was measured by incorporating 1 μCi [^3^H]thymidine (Amersham Pharmacia Biotech) per well during the last 24 h of a 3-day culture. Cells were then harvested onto a glass-fiber filter, using a Cell Harvester (Skatron Instruments), and radioactivity was estimated in counts per minute (cpm). Results are expressed as fold proliferation (= cpm_infected_/cpm_non-infected_).

### Hematological samples

Blood samples were collected at 20 weeks p.i, as above mentioned, in vials containing ethylene-diamine-tetracetic acid (EDTA) as anticoagulant. Total red blood cell (RBC) count (M/μl), hemoglobin (HGB) (g/dl), hematocrit (HCT) (%), mean corpuscular volume (MCV) (fL), mean corpuscular hemoglobin (MCH) (pg), mean corpuscular hemoglobin concentration (MCHC) (g/dl), red cell distribution width-standard deviation (RDW-SD) (fL) and red cell distribution width-coefficient of variation (RWD-CV) (%) were calculated using an automatic blood analyzer (Sysmex XE-2100). The percentage of eosinophils (EOS%) was calculated through thin blood films stained with Giemsa. At least 100 cells were counted on each film.

### Statistical analyses

Splenic and thymic cytokine expression levels (*Il4*, *Arg1*, *Ifng*, *Nos2*, *Il17*, *Foxp3*, *Il10*, *Tgfb*, *Ebi3* and *Il12a* and *IL12b*) in each rat of the NI, PI, R8 and R12 groups were compared with each other by the non-parametric Mann–Whitney test (SPSS v.22). The bivariant correlation (Kendall’s Tau-b correlation) of splenic and thymic cytokine expression levels (*Il4*, *Arg1*, *Ifng*, *Nos2*, *Il17*, *Foxp3*, *Il10*, *Tgfb*, *Ebi3* and *Il12a* and *IL12b*) vs parasitic burden, rat body weight at the end of the experiment and hematological parameters (RBC, HGB, HCT, MCV, MCH, MCHC, RDW-SD, RWD-CV and EOS%) in each rat groups (NI, PI, R8 and R12) were calculated. Statistical comparisons of the percentage of rats with anemia were carried out with the chi-square test (SPSS v.22). Principal component analysis (PCA) was applied to each individual rat data set (NI, PI, R8 and R12) to gain an overview of the degree of differentiation between the NI, PI, R8 and R12 groups. Analyses were carried out using CLIC package version 97 [37], which is freely available at http://mome-clic.com. Since PCA is an unsupervised method and makes no assumption as to the origin or class of samples, it allows the major sources of variance in a data set to be defined without incorporating an inherent bias. PCA reduces the multivariate data to a lower-dimensionality score plot, without requiring any earlier class information and delivers a snapshot of the similarity between observations based on the linear combinations of the data. As the first step, PCA components of spleen cytokine expression were calculated. Then, PCA components including parasitic burden, rat body weight at the end of the experiment, RBC, HGB, HCT, MCV, MCH, MCHC, RDW-SD, RWD-CV and EOS% in each rat group (NI, PI, R8 and R12) were calculated. Relative risks of presence of anemia (RR) were estimated using a multivariate logistic regression [in the context of risk factors, the resulting Exp(B) are estimates of RR] using SPSS v.22. Five multiple logistic regression models were analyzed. Inclusion of two terms representing multiplicative interaction between *Ifng* and *Nos2* (*Ifng* x *Nos2*) was found to be helpful in order to increase the fits (models 2–5). The following were used as independent variables: presence/absence of reinfection of liver flukes (reinfection) in model 1; reinfection, gene expression level of *Ifng*, *Nos2* and *Tgfb* in model 2; reinfection, *Ifng*, *Nos2* and *Il10* in model 3; reinfection, *Ifng*, *Nos2* and *Foxp3* in model 4; reinfection, *Ifng*, *Nos2* and *Ebi3* in model 5. Results were considered statistically significant when P < 0.05.

## Results

### *Fasciola hepatica* development in primary infection and reinfection

All rats were analyzed in the advanced chronic phase 20 weeks after initiation of the experiment (see Fig 1), harboring the following fluke burdens: i) 2.8±1.9 worms/rat (range 1–7 worms/rat) in the primary infection group; ii) 4.6±2.4 worms/rat (range 1–9 worms/rat) in the R8 reinfection group and iii) 3.1±1.6 worms/rat (range 2–6 worms/rat) in the R12 reinfection group.

### Immune response in the advanced chronic phase in primary and secondary infections

**Fig 2:**
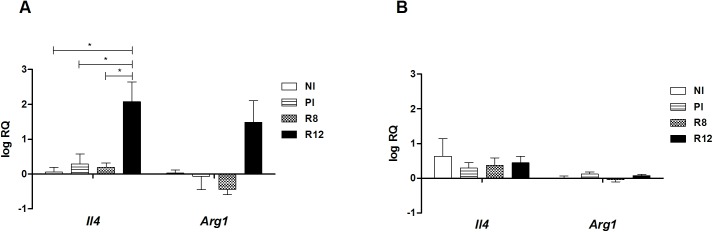
Effect of *Fasciola hepatica* reinfection on the *Il4* and *Arg1* expression from the spleen and thymus. A) Spleen. B) Thymus. Group NI: non-infected controls; Group PI: rats received a single infection dose; Group R8: infected and reinfected rats at 8 weeks after primary infection; Group R12: infected and reinfected rats at 12 weeks after primary infection. P <0.05 was considered statistically significant. Bars represent mean ± SE values. *Statistically significant differences (P<0.05, Mann-Whitney non-parametric test).

**Fig 3:**
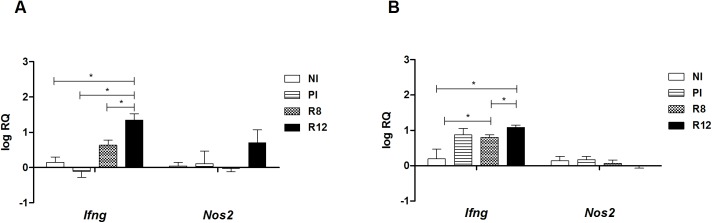
Effect of *Fasciola hepatica* reinfection on the *Ifng* and *Nos2* expression from the spleen and thymus. A) Spleen. B) Thymus. Group NI: non-infected controls; Group PI: rats received a single infection dose; Group R8: infected and reinfected rats at 8 weeks after primary infection; Group R12: infected and reinfected rats at 12 weeks after primary infection. P <0.05 was considered statistically significant. Bars represent mean ± SE values. *Statistically significant differences (P<0.05, Mann-Whitney non-parametric test).

**Fig 4:**
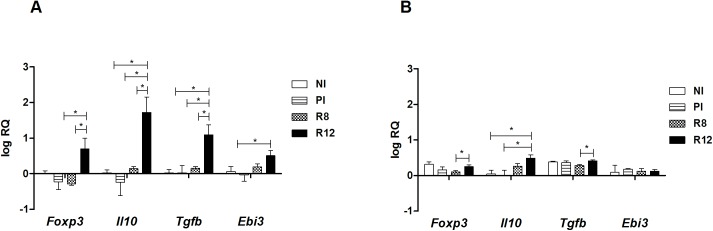
Effect of *Fasciola hepatica* reinfection on the *Foxp3*, *Il10*, *Tgfb* and *Ebi3* expression from the spleen and thymus. A) Spleen. B) Thymus. Group NI: non-infected controls; Group PI: rats received a single infection dose; Group R8: infected and reinfected rats at 8 weeks after primary infection; Group R12: infected and reinfected rats at 12 weeks after primary infection. P <0.05 was considered statistically significant. Bars represent mean ± SE values. *Statistically significant differences (P<0.05, Mann-Whitney non-parametric test).

**Fig 5:**
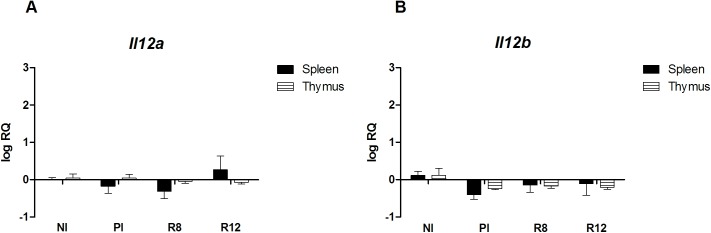
Effect of *Fasciola hepatica* reinfection on the *Il12a and Il12b* expression from the spleen and thymus. A) Spleen. B) Thymus. Group NI: non-infected controls; Group PI: rats received a single infection dose; Group R8: infected and reinfected rats at 8 weeks after primary infection; Group R12: infected and reinfected rats at 12 weeks after primary infection. (A) *Il12a* expression. (B) *Il12b* expression. Bars represent mean ± SE values. No statistically significant differences were found (P<0.05, Mann-Whitney non-parametric test).

To address the immunological status of the different infected rat groups, the expression of Th1/Th2 markers in primary and secondary infections was analyzed in the spleen and the thymus. The spleen and thymus cells of the Wistar rats were chosen as primary and secondary lymphoid organs where the immunological responses take place and different types of Tregs develop. In primary infected rats at 20 w.p.i., *Il4* and *Arg1* mRNA levels in the thymus and spleen presented basal levels, and no induction of *Ifng Nos2* was detected (Figs 2 and 3). An increase of *Il4*, *Arg1* and *Ifng* mRNAs in the spleen was observed in the R12 group, suggesting the existence of peripheral systemic mixed Th1/Th2 active immune responses in secondary infection (Figs 2 and 3). Moreover, in the spleen of R12 rats, the expression levels of *Foxp3*, *Il10* and *Tgfb* mRNAs increased in comparison with NI rats and PI rats (Fig 4). Increased expression levels of *Ebi3*, that codifies IL-27β (part of the IL-35 complex), were observed in the spleen of R12 when compared to NI rats (Fig 4). The *Ifng* expression in the thymus increased above basal levels in reinfected rats (R8 and R12 groups), indicative of an ongoing Th1 response (Fig 3). In addition, the expression levels of *Il10* in the R12 group increased in the thymus when compared to NI and PI rats (Fig 4). In primary infected rats, R8 and R12, *Il12a* and *Il12b* levels from the thymus and spleen presented basal levels (Fig 5).

**Fig 6:**
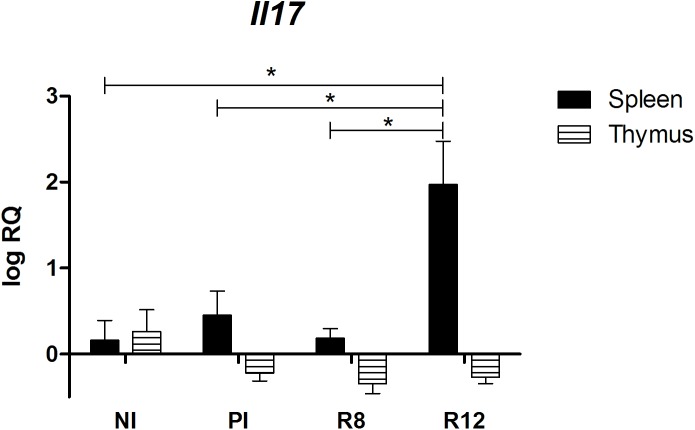
Effect of *Fasciola hepatica* reinfection on the *Il17* expression from the spleen and thymus. Group NI: non-infected controls; Group PI: rats received a single infection dose; Group R8: infected and reinfected rats at 8 weeks after primary infection; Group R12: infected and reinfected rats at 12 weeks after primary infection. Bars represent mean ± SE values. *Statistically significant differences (P<0.05, Mann-Whitney non-parametric test).

Notably, the expression of *Il17* mRNA in R12 in the spleen, but not in the thymus, increased when compared to control rats, PI and R8 rats (Fig 6).

Statistically significant positive correlations were obtained between splenic *Ifng* vs *Foxp3* (r = 0.56), *Tgfb* (r = 0.66), *Il10* (r = 0.77), *Ebi3* (r = 0.65) and *Il4* (r = -0.75). Statistically significant positive correlations were also obtained between thymic *Ifng* vs *Foxp3* (r = 0.76), *Tgfb* (r = 0.83), *Il10* (r = 0.50) and *Ebi3* (r = 0.57). A significant negative correlation was obtained between splenic *Ifng* vs *Il12b* (r = −0.61). Interestingly, a significant negative correlation between thymic *Il17* vs fluke burden (r = -0.54) was detected.

### Suppression of proliferation in the advanced chronic phase

**Fig 7:**
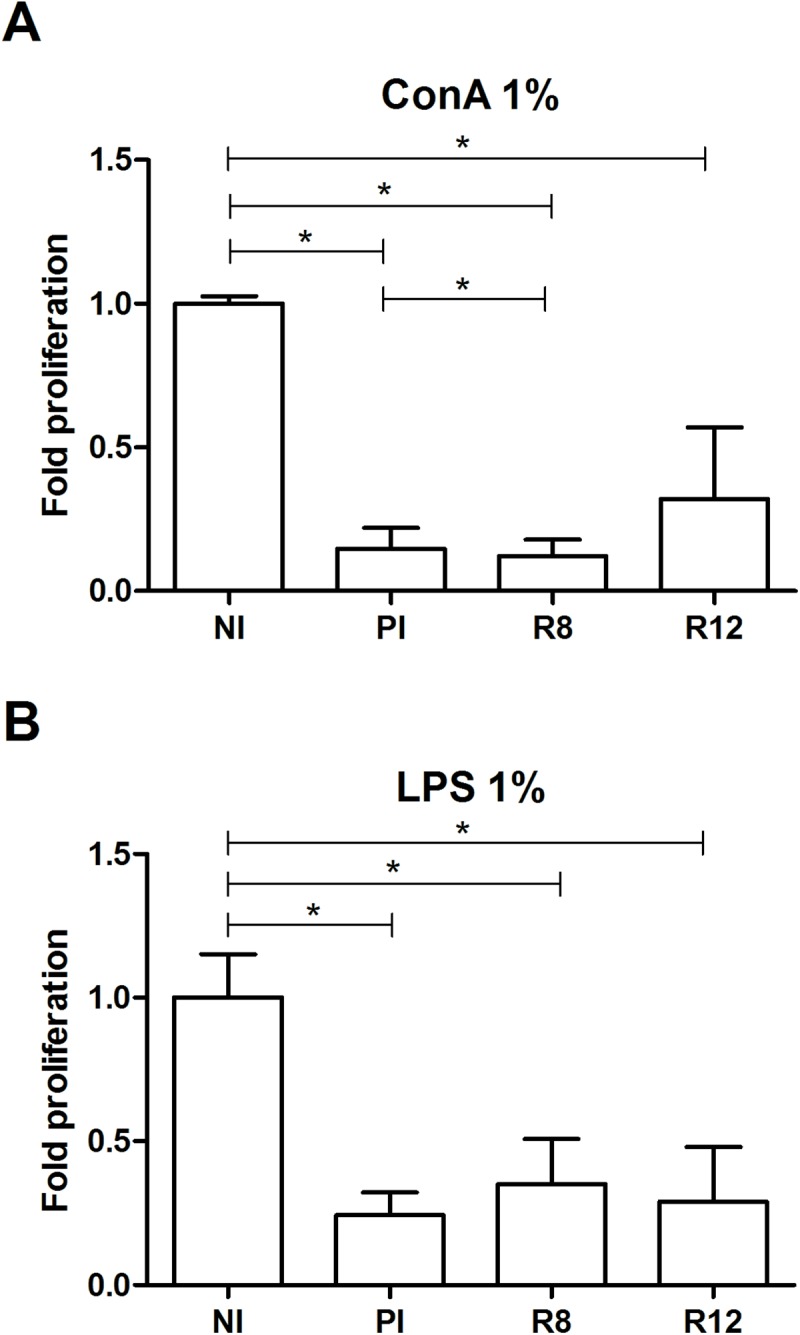
Effect of *Fasciola hepatica* reinfection on spleen cell proliferation in response to mitogens. A) Concanavalin A (ConA). B) Lipopolysaccharide (LPS). Group NI: uninfected controls; Group PI: rats received a single infection dose; Group R8: infected rats and challenged at 8 weeks after primary infection; Group R12: infected rats and challenged at 12 weeks after primary infection. Significant inhibition of the proliferation were observed in cultures stimulated with concanavalin A (ConA) and lipopolysaccharide (LPS). P < 0.05 was considered to be statistically significant. Bars represent mean ± SD. *Statistically significant differences (P<0.05, Mann-Whitney non-parametric test).

The proliferative response of lymphocytes to mitogens in the spleen was evaluated. LPS stimulates mostly B lymphocytes, but a significant decrease in proliferation (around 50%) was observed in PI, R8 and R12 rats, compared with spleen cells of NI (*P* < 0.05) (Fig 7). When T cells were stimulated with ConA, a T cell mitogen, a profound decrease in the proliferation of spleen cells from PI, R8 and R12 compared with spleen cells of NI rats was also found (*P* < 0.05) (Fig 7). These results suggest that a pronounced suppression of a mitogen-induced proliferative response of lymphocytes takes place in all animals, whether primo-infected or reinfected during the advanced chronic phase.

### Pathogenicity and systemic cytokine expression levels

**Fig 8:**
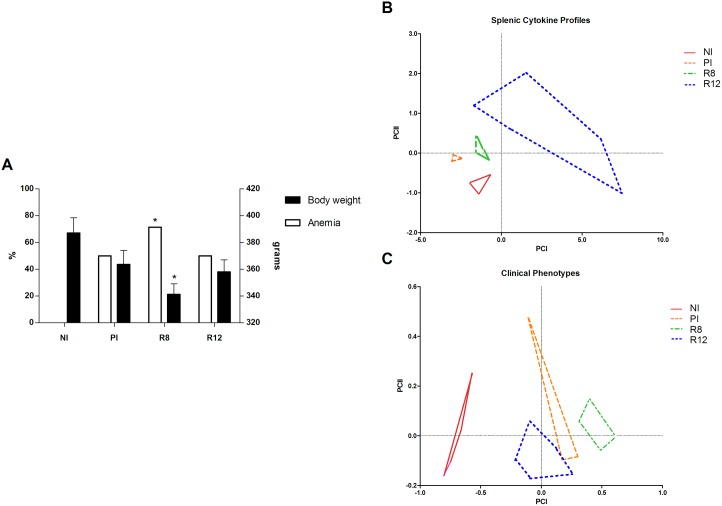
Pathogenicity and systemic cytokine expression levels. A) Effect of *Fasciola hepatica* reinfection on anemia and rat body weight. B) Factor map corresponding to splenic cytokine profiles from experimentally infected Wistar rats including fluke burden. Samples are projected onto the first (PCI, 86%) and second (PCII, 5%) principal components. Each group is represented by its perimeter. C) Factor map corresponding to clinical phenotypes from experimentally infected Wistar rats. Clinical phenotypes are defined by fluke burden, rat body weight, RBC, HGB, HCT, MCV, MCH, MCHC, RDW-SD, RWD-CV and EOS% detected in each rat group (NI, PI, R8 and R12). Samples are projected onto the first (PCI: 84%) and second (PCII: 11%) principal components. Each group is represented by its perimeter. Group NI: non-infected controls; Group PI: rats received a single infection dose; Group R8: infected and reinfected rats at 8 weeks after primary infection; Group R12: infected and reinfected rats at 12 weeks after primary infection. Bars represent mean ± SE values. *Statistically significant differences (rat body weight: Mann-Whitney non-parametric test; anemia: chi-square test; P<0.05).

Rat body weight, RBC, HGB, HCT, MCV, MCH, MCHC, RDW-SD, RWD-CV and EOS% in each rat group (NI, PI, R8 and R12) were calculated at the end of the experiment (Table 1). Anemia was defined on the basis of the hemoglobin cut-off values of hemoglobin mean–S.D. of the control rats [38]. An increase in the percentage of the number of anemia cases was observed in the R8 group vs NI (chi-square: 5.25, P<0.05) (Fig 8A). Additionally, the rat body weight in R8 rats was lower than in negative control rats (NI) (Fig 8A).

**Table 1:** Rat body weight and hematological parameters in each rat group at the end of the experiment.

	NI	PI	R8	R12
**Body weight**	0.41 ± 0.02	0.35 ± 0.01	0.33^a^ ± 0.01	0.35 ± 0.02
**RBC**	10.22 ± 0.41	7.76^a^ ± 1.82	6.16^a^ ^b^ ± 0.30	7.11^a^ ± 0.89
**HGB**	18.33 ± 0.73	13.93^a^ ± 4.83	10.90^a^ ± 1.67	13.08^a^ ± 2.17
**HCT**	52.05 ± 0.86	43.00^a^ ± 7.54	37.06^a^ ^b^ ± 4.15	41.62^a^ ± 4.97
**MCV**	51.00 ± 1.40	56.00^a^ ± 5.12	60.16^a^ ± 5.66	58.63^a^ ± 2.95
**MCH**	17.95 ± 0.29	17.70 ± 2.03	17.94^a^ ± 2.32	18.37 ± 1.55
**MCHC**	35.20 ± 1.10	31.80 ± 5.20	29.78^a^ ± 1.46	31.30 ± 1.64
**RDW-SD**	25.58 ± 1.72	41.27^a^ ± 11.58	51.14^a^ ^b^ ± 10.11	36.08^a^ ^c^ ± 6.91
**RWD-CV**	17.90 ± 0.50	22.83 ± 1.68	24.38^a^ ± 4.45	18.70^c^ ± 2.84
**EOS%**	1.43 ± 1.26	6.33 ± 3.21	8.22^a^ ± 1.28	3.45^c^ ± 0.98

Values are means ± S.D. **NI**, uninfected controls; **PI**, rats received a single infection dose; **R8**, infected rats and challenged at 8 weeks after primary infection; **R12**, infected rats and challenged at 12 weeks after primary infection; **Body weight**, rat body weight (gr); **RBC**, total red blood cell count (M/μl); **HGB**, hemoglobin (g/dl); **HCT**, hematocrit (%); **MCV** mean corpuscular volume (fL); **MCH**, mean corpuscular hemoglobin (pg); **MCHC**, mean corpuscular hemoglobin concentration (g/dl); **RDW-SD**, red cell distribution width-standard deviation (fL); **RWD-CV**, red cell distribution width-coefficient of variation (%); **EOS%**, percentage of eosinophils (%).

^a^ Significantly different vs. NI, determined by Mann-Whitney test.

^b^ Significantly different vs. PI, determined by Mann-Whitney test.

^c^ Significantly different vs. R8, determined by Mann-Whitney test.

The gene expression levels obtained in cytokines analyzed from the thymus and spleen in each rat group and its clinical phenotype, as defined by rat body weight, RBC, HGB, HCT, MCV, MCH, MCHC, RDW-SD, RWD-CV and EOS% were correlated to elucidate model-dependent associations of the cytokine gene expression and pathogenicity. A significant positive correlation (Tau b de Kendall) between: i) *Ifng* expression levels and MCV (r = 0.503, P<0.05); ii) *Il17* expression levels and RBC (r = 0.474, P<0.05), HCT (r = 0.490, P<0.05) and MCHC (r = 0.613, P<0.05); iii) *Arg1* expression levels and RBC (r = 0.482, P<0.05), HGB (r = 0.572, P<0.05) and HCT (r = 0.455, P<0.05) were detected.

The RR of anemia associated with reinfection (Table 2) showed a value of 14.66 (model 1). The dependence of anemia in reinfection on the combination of *Ifng* and *Nos2* was analyzed through multiple logistic regression analysis. Model 2 showed an anemia risk in the covariable *Ifng* x *Nos2* of 50.28, but the covariable *Tfg* presented a RR value of 0.006, indicating a protective value. Model 3 showed an anemia risk in the covariable *Ifng* x *Nos2* of 37.32, but the covariable *Il10* presented an RR value of 0.05, indicating a protective value. Models 4 and 5 included as covariables *Ifng* x *Nos2* and *Foxp3* (model 4), and *Ebi3* (model 5) but presented no significant P values. These results indicate that increased systemic *Tgfb* and *Il10* expression levels decrease the anemia risk associated with inflammation.

**Table 2:** Multivariate logistic regression analysis of different factors obtained in the course of experimental fascioliasis in Wistar rats and regression coefficients [Exp(B) = RR = relative risk of presence of anemia] with significance in different models.

		Reinfection	*Ifng* x *Nos*2	*Tgfb*	*Il10*	*Foxp3*	*Ebi3*
**Model 1**	**RR**	14.66					
	**P-value**	0.009					
**Model 2**	**RR**	1010.65	50.28	0.006			
	**P-value**	0.003	0.047	0.037			
**Model 3**	**RR**	564.72	37.32		0.05		
	**P-value**	0.002	0.043		0.017		
**Model 4**	**RR**	32.09	8.00			0.07	
	**P-value**	0.001	NS			NS	
**Model 5**	**RR**	32.72	2.57				0.17
	**P-value**	0.002	NS				NS

**Reinfection**, presence/absence of reinfection of liver flukes; ***Ifng***, gene expression level of Interferon gamma; ***Nos2***, gene expression level of inducible nitric oxide synthases; ***Tgfb***, gene expression level of transforming growth factor beta; ***Il10***, gene expression level of interleukin 10; ***Foxp3***, gene expression level of Forkhead Box P3; ***Ebi3***, expression level of Epstein-Barr virus-induced gene 3, a subunit of interleukin 35; ***Il17***, gene expression level of interleukin 17; ***Il4***, gene expression level of interleukin 14; ***Arg1***, gene expression level of arginase 1. NS = not significant.

5 models (model 1, 2, 3, 4 and 5) were used in the multivariate logistic regression analysis including presence/absence of anemia as dependent variable: **model 1** including presence of reinfection as independent variable; **model 2** including presence of reinfection, *Ifng*, *Nos2* and *Tgfb* as independent variables; **model 3** including presence of reinfection, *Ifng*, *Nos2* and *Il10* as independent variables; **model 4** including presence of reinfection, *Ifng*, *Nos2* and *Foxp3* as independent variables; **model 5** including presence of reinfection, *Ifng*, *Nos2* and *Ebi3* as independent variables.

### Multivariate statistical modeling of *F*. *hepatica* reinfection

The fluke burden and the splenic and thymic cytokine profiles detected in each rat group (NI, PI, R8 and R12) were modeled through principal component analysis (PCA) (Fig 8B). In brief, only in the splenic cytokine profiles, there are clear visual separations among the NI, PI, R8 and R12 groups.

The fluke burden and the clinical phenotypes, as defined by rat body weight, RBC, HGB, HCT, MCV, MCH, MCHC, RDW-SD, RWD-CV and EOS% detected in each rat group (NI, PI, R8 and R12) were modeled through principal component analysis (PCA) (Fig 8C). In brief, clinical phenotypes can be differentiated among the NI, PI, R8 and R12 groups (PCI: 84%, PCII: 11%).

## Discussion

The host’s ability to develop resistance to *Fasciola* reinfection is species-dependent. Thus, in sheep sensitization to a primary *F*. *hepatica* infection fails to stimulate any resistance to secondary infection [39]. Previous studies showed that rats exhibit significant resistance to reinfection [40,41], depending on the strain, age, and sex [41–46]. Rats infected orally with metacercariae quickly build up resistance to a secondary challenge within 2–3 weeks before the majority of flukes reach the liver [41]. Previous studies related the Th2 response to the protection capacity in rats [44–60].

Wistar rats are frequently used as a laboratory model for chemotherapeutic, pathological and immunological studies and are especially useful for investigations during the advanced chronic period of fascioliasis [3,6,34,35]. Also, it is accepted that primary infections cause partial protection against a secondary reinfection in some rat strains, the so-called concomitant immunity [61–63], which is the reason why rats are presently not used in models of experimental reinfection. *Fasciola* reinfection was confirmed in Wistar rat/*F*. *hepatica* reinfection models focusing on IgG and IgA serum levels and coproantigens. These studies showed different kinetics of the humoral response depending on whether rats were challenged in the acute phase or in the chronic phase [62].

The various stages of the *F*. *hepatica* life cycle may contribute to heterogeneity in immune responses. Juvenile migrating flukes, adult flukes in the bile duct and eggs elicit stage-specific immune responses [27,64] that may change over time in the infected host. Previous studies [18,65] suggest that two different forms of immunity are present in parasite life cycles which imply more than one location in the host, i.e., a migratory pattern is adopted within host tissues [66]. In a bovine/*F*. *hepatica* model, it has been found that both TGF-β and IL-10 play a decisive role in the control of IL-4 and IFN-γ-dependent anti-parasitic pathways, suggestive of various regulator mechanisms which may be limiting the host immune response [31].

We previously found that rats infected with *F*. *hepatica* experience immunosuppression during the chronic phases of the disease [35]. This immunosuppression may allow parasite survival when confronted with an ongoing immune response during primoinfection, and it is likely to be caused by Th2 cytokines as IL-4 that inhibit Th1 and Th17 responses as well as Tregs that produce suppressive cytokines as IL-10 and TGF-β. Thus, a polarized Th2/Treg immune response coincident with a suppression of Th1/Th17 cytokines is generated during primoinfection, having been previously described in other experimental models [22–26,31,67–69].

In the present study, we observed no significant differences in thymus or spleen Th1, Th2, Th17 and Treg associated gene expression markers in primoinfected rats *vs* controls at 20 w.p.i., indicating that in the advanced chronic phase of the disease (20 w.p.i.), those specific alterations of the expression pattern of the immune response due to primary infection return to normal levels. However, upon reinfection, a different immune response expression pattern emerges, likely to represent a secondary/memory expansion of the immune response. *Ifng* gene expression increased in the thymus of R12 rats with respect to non-infected rats. More notably, in R12 rats, in addition to *Ifng*, splenic expression levels of numerous cytokines and *Foxp3* increased. At this time of reinfection, full establishment or adult flukes in the bile duct is achieved. These data indicate that reinfection increases *Ifng* gene expression levels. Previously, a mixed Th1/Th2 response was demonstrated in given cases in *F*. *hepatica* primoinfection, i.e., depending on the genetic background of some species as, for example, in the C57BL/6 mice/*F*. *hepatica* model [25].

In Wistar rats, IL-4 has been demonstrated to be a characteristic element of the Th2/Th0 response associated with *F*. *hepatica* infection [70,71]. Previous studies performed on T helper cytokines in rats basically concerned the early stages of fascioliasis, suggesting that *Fasciola* species, similar to other helminth parasites, induce a polarized Th2 response [70,71]. The rise in levels of parasite-specific IL-4 after TGF-β inhibition suggests that TGF-β may be responsible for inhibiting IL-4-dependent anti-parasitic mechanisms during acute infection, such as eosinophilia or mastocytosis. Migrating juvenile worms would be more susceptible to IL-4-dependent cellular infiltration, as other models of IL-4-dependent worm immunity have demonstrated [72]. We previously described that in primoinfected animals that reach the early chronic infection, there is a predominance of Th2 response, which decreases in advanced chronic infection, at 20 w.p.i., which is characterized by a persistent immune suppression [35].

During the chronic stage of the disease, the R12 group showed, in the thymus, a progression of host responses from effector Th2 to a so-called ‘modified Th2’ phenotype associated with elevated Treg-associated gene expression levels (Il10) and reduced Th2 gene expression levels but with increased Ifng expression. The absence of *Foxp3* and *Tgfb* expression in the thymus in the R12 group might indicate that the source of *Il10* in the thymus could be IL-10-producing-Foxp3-negative cells [73] either Th1, Th2 and Th17 cells, thus given the increased *Ifng* levels, or Th1 cells are the ones in which *Il10* is increased. Alternatively, these cells could be Tr1 cells that require antigen for IL-10 secretion [16].

In the spleen, the R12 group showed an even higher increase in the levels of *Ifng*, *Il10*, *Tgfb* and *Foxp3*, and in addition, increased levels of *Ebi3* (IL-27β component of IL-35), indicating an expansion of Tregs. Tregs may be responsible for the increase in *Il10*, *Tgfb* and *Ebi3* levels, inhibiting the production of Th2 cytokines, but allow a partial Th1 response (evidenced by *Ifng* expression) in situations of reinfection. Moreover, significant positive correlations between splenic *Ifng* vs *Foxp3*, *Tgfb* and *Ebi3* were detected, suggesting that the role of Treg is highly selective, and does not suppress the Th1 response observed upon reinfection at 12 w.p.i. Such an immune response representing a regulated Th2 response may be an important feature of balanced parasitism that ensures parasite survival but protects the host from Th2-induced pathology. Previous research on the protection in human schistosomiasis has also emphasized the compartmentalization of the type-1⁄2 immune response against different parasite stages, and the need for more detailed *in vivo* investigations into immune mechanisms at different sites of infection [20]. In the early stage of primoinfection, Tr1-type clones generated from mice infected with *Fasciola* suppressed proliferation and IFN-γ production by Th1 cells [32]. IFN-γ was also associated with resistance against the liver stage in human schistosomiasis [20]. Previous studies have demonstrated that an early and local type-1 immune response is associated with the resistance of Indonesian thin-tail (ITT) sheep to *Fasciola* infection [19].

The type-1 immune response was highlighted in the hepatic lymph nodes (HLN) of *F*. *gigantica*-infected ITT sheep (resistant infection) and was in direct contrast to the predominant type 2-like response observed in *F*. *hepatica*-infected ITT sheep (susceptible infection) and *F*. *gigantica*-infected Merino sheep (susceptible breed). The significant type-1 immune response occurred at the time when parasite attrition occurs and may represent the immune phenotype responsible for resistance against *Fasciola* infection in the natural host [19].

In other helminth infections, Tregs play a beneficial role for the parasite, as in experimental rodent filariasis, *S*. *mansoni* or *Litosomoides sigmodontis* infection, being also beneficial to the host by reducing the associated immunopathology [74,75]. Interestingly, *Brugia malayi* secretes a homolog of mammalian TGF-β [76] that may be involved in Treg cell conversion. It has been demonstrated that increased IFN-γ production, by IL-10 inhibition or knockout, results in more effective killing of *T*. *spiralis* larvae located within the muscles of murine hosts. Conversely, in the same IL-10^-/-^ mice, IL-4 and IL-13 were boosted but mast cell infiltration was reduced, leading to increased worm burdens within the intestinal tract [65]. In *Litomosoides sigmodontis*-infected mice it has been demonstrated that the restoration of effective parasite clearance only occurred when surface bound CTLA-4 was neutralized and CD4+CD25+ cells were depleted simultaneously [77]. In a *T*. *spiralis/*mice model, it was found that the muscle infection parasite killing happened in the presence of increased IFN-γ and increased infiltration of inducible nitric oxide synthase (iNOS)-positive cells [18].

In our study, induction of Treg markers in R12 was paralleled by the induction of *Il17*, in which several pro-inflammatory effects have been described [78]. However, it also plays a protective role against pathogens such as *Trypanosoma cruzi* [79]. In this sense, the negative correlations detected between *Il17* levels in the spleen vs the phenotypic parasitic markers used in this work, i.e. number of liver flukes detected in the bile duct, suggest a protective role of the IL-17 and Th17 response previously unappreciated in fascioliasis, but described in other helminthic infections in association with a Th2 response.

Interestingly, in endemic schistosome and filarial diseases, pathology ranges from chronic debilitating to tolerant phenotypes. The pathologic phenotype is characterized by a Th2 primary response that converts to Th1 and Th17 in secondary infection, while the tolerant phenotype shows a regulatory response, low pathology but high parasite burden [80]. Thus, in the chronic phase in endemic areas prone to reinfection, the response is expected to be more complex, involving several types of immune response as our results show here.

In addition, it is controversial whether regulatory T cells under certain conditions may express pro-inflammatory cytokines while retaining suppressive ability, or whether these Tregs are dysregulated and associated with perpetuation of the immunopathology.

Moreover, induction of alternative activated macrophages (AAMφ), dependent on IL-4, has been described at the early stages of *F*. *hepatica* infection [81], which can also be induced in mice through the injection of excreted/secreted products of *F*. *hepatica* that induce the recruitment of alternatively activated macrophages leading to suppressed *Nos2* mRNA levels and elevated *Tgfb* levels [22]. In turn, AAMφ produce molecules that are toxic to the fluke and participate in fibrosis and tissue repair [82]. In cattle [83] and sheep [84], it was found that IL-10 and TGF-β levels were increased upon *F*. *hepatica* infection. These results lead to the hypothesis that IL-10 and TGF-β worked together, being responsible for fibrosis and ensuing parasite burden decrease. Such previous observations are in agreement with our results in the R12 rats due to the increase in *Arg1* levels in the spleen. A similar trend was observed for IL-10 and TGF-β. Thus, during reinfection AAMφ could be reactivated and is likely to result in reduced proliferation in spleen cells through IL-10 and TGF-β. In addition, AAMφ could participate in tissue repair.

In rats, sheep, and humans, *F*. *hepatica* excretory/secretory antigens (ESFh) have been shown to inhibit the proliferative response of spleen mononuclear cells stimulated with mitogens in a dose-dependent fashion [85]. Previous studies carried out at our laboratory already documented spleen cell immunosuppression in the advanced chronic phase of fascioliasis [35]. A decrease in nitric oxide production by lipopolysaccharide (LPS)–stimulated peritoneal macrophages has also been observed with ESFh, although the exact mechanism was not addressed [86]. Tregs have been described to cause immunosuppression in other parasitic diseases. In our study, spleen cell unresponsiveness to B and T mitogens is present in primo-infected and re-infected groups. Thus, immunosuppression is present in the advanced chronic phase independent of reinfection. It has been described that in the migration phase of its life-cycle, *F*. *hepatica* generates a transient immunosuppression. Thus, when evaluating the spleen cell proliferative response to ConA of *F*. *hepatica*-infected rats until 60 days post-infection, a decrease on day 7 post-infection was shown, while LPS caused a pronounced increase of the proliferative response from day 3 until around 8 weeks post-infection [28,86]. In addition, in a sheep model, after secondary infection at 6 weeks post-infection, ConA-induced lymphocyte proliferation transiently increased, while the humoral response was reduced [39]. The main difference between those studies and ours is that we measured proliferation much later, at 20 w.p.i., while in previous studies it was carried out at 9 w.p.i. at the most. Our results show that a generalized unresponsiveness to T and B cell mitogens is preserved along the infectious phases up to the advanced chronic phase independently of reinfection. However, the PI and R8 groups did not show changes in *Il10* nor *Tgfb* expression in the spleen, suggesting that suppression of spleen cell proliferation by parasite molecules might be occurring. But, in the R12 group, a partial Th1 response as well as Treg and Th2/M2 cytokine responses in the spleen occurred. Thus, in R12 rats in addition to parasite immunosuppressive antigens, host immunoregulation might occur, and in R8 rats bystander suppression of *Ifng* might occur probably due to the location of the parasite in the bile duct. Furthermore, Treg markers, *Foxp3*, *Il10*, *Tgfb* and *Ebi3*, reached high levels of gene expression that are likely to correspond to Th3 iTreg cells. Notably, our results suggest, for the first time, that IL-35, likely to be produced by Tregs, may play a suppressive role in *Fasciola* reinfection.

The association between fascioliasis-induced anemia and related factors has previously been quantified in a rodent model [3]. The development of anemia appears to be complex and may involve multiple mechanisms. Among the mechanisms that explain fascioliasis-related anemia are: i) compensatory increase in erythrocyte production and a continuous drain of iron stores resulting from the blood-sucking activity of the parasites; ii) hemolysis of red blood cells; iii) the general effects of inflammation on erythropoiesis; iv) concomitant parasitic and bacterial infections, and v) premorbid nutritional abnormalities [3]. In this study, the splenic and thymic cytokine expression levels in F. hepatica reinfections were correlated with the anemia clinical phenotype. Herein, experimental infections in an animal model of chronic and repeated fascioliasis infection, related to the expression levels of Th1/Th2/Th17/Treg with anemia during re-exposure have been carried out for the first time. The increased RR of anemia when *Ifng* expression levels were included as covariables in the models analyzed are suggestive of the presence of anemia of inflammation. In addition, it was shown in murine *Toxoplasma gondii* infection that IFN-γ can act directly on macrophages to provoke RBC uptake [87]. The results of logistic regression analysis suggest that increased splenic *Tgfb* and *Il10* expression levels are protective in anemia of inflammation. Although inflammation is fundamental to immune-mediated protection against most pathogens, counter-regulatory mechanisms are required to limit collateral damage to host tissues [88]. Our results suggest that anemia of inflammation is present in re-infection. Similarly, anemia in patients with schistosomiasis is usually anemia of inflammation, linked with blood loss, which contributes to total-body iron deficiency [89]. Mechanistically, anemia of inflammation is mainly caused by inflammation-driven retention of iron in macrophages making the metal unavailable for heme synthesis in the course of erythropoiesis and further by impaired biological activity of the blood cell hormone erythropoietin and the reduced proliferative capacity of erythroid progenitor cells, i.e. anemia of inflammation is caused by iron trapping with the body mediated by the hepatic hormone hepcidin, the release of which is stimulated by infection-related production of the pro-inflammatory cytokine interleukine 6 [90]. CD4+CD25+Foxp3+ regulatory cells (Tregs) are a special lineage of cells, essential for maintaining immune homeostasis. They are conventionally associated with the production of classical anti-inflammatory cytokines such as IL-10, TGF-b and IL-35, and are consistent with their anti-inflammatory functions. Herein, *Il10*, *Tgfb* and *Il35* gene expression levels were evaluated. Concretely, our results suggest that Tregs participate in the regulation of anemia of inflammation. The results showed that in reinfection two phenotypic patterns were detected: i) a pattern which included only increased systemic *Ifng* expression levels but no Treg associated gene expression, associated with lower hemoglobin levels; ii) a pattern which included increased systemic *Ifng* expression levels as well as an increased Treg gene expression associated with a less severe decrease of hemoglobin levels. These results agree with previous data in which TGF-β production is inversely correlated with severity of murine malaria infection, suggesting that TGF-β may play a crucial role in preventing the severe pathology of malaria [91]. We hypothesized that the severe anemia of fascioliasis infection in the R8 group was due to *Ifng* increased expression in the absence of *Tgfb* or *Il10*. Examples of the role of Treg in reducing immunopathology can be found in the original observation of their role in control of colitis [92], reduction of pulmonary inflammation in pneumocystis [93], control of hepatic pathology in *Schistosoma* infections [94] or control of immunopathological lesions in viral infections [95].

## Conclusions

Depending on whether adult flukes are well established in the bile duct of primary infected rats or not, it has been demonstrated that reinfection generates a strong or a mild immune response, respectively. Thus, in animals with established *F*. *hepatica* infection a huge increase in the immune response takes place, which is a mixed Th2/Treg associated gene expression together with *Ifng* expression. Interestingly, a Th17 associated gene expression, which is inversely correlated with reinfection, can also be observed, being suggestive of a protective effect of this response that deserves further investigations. Immune suppression is maintained from primo-infection to chronic infection, and also in reinfection. The systemic immune response is different in each group, suggesting that suppression is mediated by different mechanisms in each case. Immune suppression could be due to the parasite in PI and R8 rats and to the induction of suppressive cells such as Treg and AAMφ in R12 rats.

The present study is the first to provide a fundamental insight into the immune profile in fascioliasis reinfection and its relation with the clinical phenotypes of anemia.
